# The molecular characteristic analysis of PRRSV GSWW/2015 strain and its pathogenicity to pigs

**DOI:** 10.1186/s12917-018-1548-3

**Published:** 2018-08-17

**Authors:** Weijie Bai, Zhijia Wang, Pu Sun, Jing Zhang, Huifang Bao, Yimei Cao, Yanyan Chang, Zaixin Liu, Dong Li, Zengjun Lu

**Affiliations:** State Key Laboratory of Veterinary Etiologic Biology, Lanzhou Veterinary Research Institute, Chinese Academy of Agricultural Sciences, Lanzhou, 730046 Gansu China

**Keywords:** PRRSV GSWW/2015 strain, Genetic variation, Pathogenicity, Viremia

## Abstract

**Background:**

Porcine reproductive and respiratory syndrome (PRRS) is a severe disease, causing great economic losses to the pig industry. The causative agent, porcine reproductive and respiratory syndrome virus (PRRSV) is highly variable. Since the emergence of highly pathogenic PRRSV (HP-PRRSV) in China in 2006, this virus strain has undergone extensive variation. To investigate the genetic variation and pathogenicity of currently isolated PRRSV GSWW/2015 strain, its whole genome was sequenced and analyzed for the specific variation in NSP2, GP3 and GP5 regions. Pigs were challenged with the isolated virus to investigate its pathogenicity.

**Methods:**

The PRRSV GSWW/2015 strain was isolated by seeding the viral material in Marc-145 cells. The virus specific cytopathic effect (CPE) was confirmed by indirect immunofluorescent assay (IFA) and PCR to detect the virus protein and RNA. Nine pairs of primers were designed to obtain the complete genome by PCR. All PCR fragments were cloned into T-vector for sequencing. The genetic variation of GSWW/2015 strain was analyzed by multiple sequence alignments. Nineteen PRRSV-free piglets were intranasally challenged with 10^8^ copies of GSWW virus, while seven piglets were housed together as contact-infected control. Clinical signs were recorded daily after challenge. Blood samples were obtained every week and the viral titer was detected by quantitative real-time PCR (qRT-PCR). The PRRSV specific antibody was detected by LSI ELISA kit.

**Results:**

The complete genome of PRRSV GSWW/2015 strain (GenBank accession number KX767091) was obtained. The whole genome of this strain shares 88.5 and 60.6% identity with VR-2332 and LV respectively, indicating that it belongs to the North American type (NA-type). Sequence alignments revealed that GSWW/2015 strain has a discontinuous deletion of 30 amino acids in NSP2, which is similar with HP-PRRSV. Some amino acids mutations can be observed in antigenic epitope regions of GP3 and GP5 compared with earlier strains of HP-PRRSV. Some piglets showed typical clinical signs of PRRSV after challenge. Only four pigs showed viremia within 3 days after challenge, most pigs showed peaked viremia after 21–28 days including 7 contact-infected pigs. Two pigs were detected to be positive for antibody to PRRSV at 14 days post infection (DPI), and 11 pigs (11/26) show seroconversion for PRRSV at 49 DPI. Twelve piglets died of PRRSV infection within two months.

**Conclusions:**

The genome of PRRSV GSWW/2015 strain shows the features of HP-PRRSV with 30 discontinuous amino acids deletion in NSP2 and some new amino acid mutations in epitope regions of GP5 and GP3, which might alter the antigenicity of the virus. Furthermore, the virus showed high virulence to piglets as reported in HP-PRRSV, and induced long-lasting viremia and low level of antibody responses. This work further enriched our knowledge on PRRSV evolution and pathogenicity.

## Background

PRRS is one of the most devastating swine diseases, which has caused enormous economic losses to global pig industry [15]. PRRS first emerged in Western Europe and North America in the 1990s and now has become an endemic disease worldwide [3, 19]. The pathogenic PRRSV mainly causes reproductive failure in sows and respiratory disorder in all-age pigs. PRRSV is an enveloped RNA virus and classified as a member of the order *Nidovirales*, family *Arteriviridae*, which also contains equine arteritis virus (EAV), lactate dehydrogenase-elevating virus (LDV) and simian hemorrhagic fever virus (SHFV) [5]. Due to the genetic and antigenic differences, PRRSV can be divided into two major genotypes: the European type (EU-type, type 1) and North American type (NA-type, type 2). Representative strains of the two genotypes are LV and VR-2332 respectively, sharing only approximately 55–70% nucleotide and 50–80% amino acid similarity [10]. In 2016, the International Committee on Taxonomy of Viruses split PRRSV into two new species defined as porcine reproductive and respiratory syndrome virus 1 (PRRSV-1) and porcine reproductive and respiratory syndrome virus 2 (PRRSV-2). The single positive-stranded PRRSV genome is approximately 15 kb in length and contains ten open reading frames (ORF): ORF1a, ORF1b, ORF2a, ORF2b, ORFs 3–5, ORF5a and ORFs 6–7 [13]. ORF1a and ORF1b encode replication-related polymerase proteins, which are cleaved into at least 16 nonstructural proteins (nsp): nsp1α, nsp1β, nsp2, nsp2NF, nsp2TF, nsp3–6, nsp7α, nsp7β and nsp8–12. The 3′-end of the viral genome contains eight ORFs encoding structural proteins, including GP2a,E, GP3, GP4, GP5, GP5a, M and N. Within PRRSV genome, nsp2 undergoes remarkable genetic variation associated with natural mutations and deletions. GP3 and GP5 are also highly variable among structural proteins. Therefore, nsp2, GP3 and GP5 are often used for phylogenetic analysis for the genetic variation and molecular epidemiology.

In 2006, a highly pathogenic PRRS (HP-PRRS) emerged in China with characteristics of high fever, increased morbidity and mortality [17]. The agent, HP-PRRSV had a unique discontinuous deletion of 30 amino acids in nsp2 and has become a dominant strain prevalent in the field. To learn the evolution of the currently circulated strains in China, one PRRSV strain named GSWW/2015 was isolated from the lung tissue of a sick pig collected from a farm in Gansu Province in 2015. The complete genome of GSWW/2015 was obtained and compared with 34 reference strains. Amino acid sequences of nsp2, GP3 and GP5 were analyzed in detail to study the genetic variation of this virus isolate. Furthermore, the pathogenicity of this virus strain was investigated by infection of piglets with cell-cultured virus material. This research provides further information on the understanding of the evolution and pathogenicity of currently circulated high virulent PRRSV.

## Methods

### Clinical samples

The pigs in one farm in Wuwei county, Gansu Province of China were suspected to be infected by PRRSV in 2015. According to the farmer’s description, some pigs showed typical clinical signs of PRRSV infection, such as high fever, blue ears, anhelation and eventual death. The lung tissue was collected from a dead pig by a local veterinarian and then sent to our lab for further confirmation of PRRSV infection. Then the lung tissue was homogenized in Dulbecco’s modified Eagle’s medium (DMEM, Gibco) for RNA extraction and virus isolation.

### RT-PCR detection of the virus RNA in viral material

Total RNA was extracted from tissue homogenates by the RNeasy Mini Kit (QIAGEN, Germany). Then the cDNA was synthesized by reverse transcription with the Superscript reverse transcriptase (Invitrogen, USA) using the oligo(dT) primer. PCR was performed using a pair of specific primers to amplify a 375 bp conserve gene fragment in M protein (forward: 5`-CCTTCGGGTACATGACATTCGT-3`; Reverse: 5`-TTGCTGCTTGCCGTTGTTATT-3`). The PCR products were analyzed by agarose gel electrophoresis to confirm the existence of virus RNA.

### Virus isolation

The tissue homogenates were centrifuged at 12,000 g for 20 min. The supernatant was passed through a 0.22-μm filter and then added on Marc-145 cell monolayer. After incubation for 1 h at 37 °C, the medium was replaced with new DMEM containing 2% fetal bovine serum (FBS, Gibco) and antibiotics (50 μg/ml penicillin, 50 μg/ml streptomycin, 100 μg/ml neomycin) at 37 °C for 3–5 days. The culture supernatant was harvested and freeze-thawed for three times when cytopathic effects (CPE) appeared. The supernatant was then transferred to Marc-145 cells for virus propagation and stored at − 70 °C as viral stocks.

### Indirect fluorescent antibody assay (IFA)

Marc-145 cells were incubated with the isolated virus for 1 h at 37 °C and then maintained in DMEM with 2% FBS and antibiotics. At 48 h post-infection, cells were washed with PBS and then fixed with methanol-acetone (1:1) for 15 min at room temperature. Cells were washed with PBS for three times and then incubated with monoclonal antibody (Mab) SR30 (RTI, 1:500) against PRRSV N protein for 1 h. After washing with PBS for 5 times, cells were incubated with FITC-conjugated goat anti-mouse secondary antibody (Molecular Probes, 1:100) for 1 h. Then cells were washed 5 times with PBS and staining was visualized with a fluorescent microscope.

### The whole genome sequencing

Nine pairs of primers were designed based on HP-PRRSV strain SHH (Accession number EU106888) to amplify the full-length genome of GSWW strain (Table 1). The PCR products were purified and cloned into pEASY-T1 vector. For each fragment, at least 3 positive bacteria colonies were selected for plasmid extraction and sequencing to get the correct genome information. Recombinant plasmids were sent to TaKaRa (Dalian, China) for sequencing.

**Table 1:** Primers for PCR of the complete genome of PRRSV GSWW/2015 strain

Fragment	Sequence of primers(5′-3′)	Position in genome
A	ATGACGTATAGGTGTTGGCTCTATG	1–1518
	GGAAATGCAGTGCCAACCACARTTC	
B	ACTTCTCYGTTAAGGAGAGTTGGAT	1175–4216
	GATACAGTCTGCAACAATGCCAAGCCTAAGCA	
C	GTTACAGTTACCCATTCTTCGG	3911–5191
	GAGGTACCCAAACATAACTAGC	
D	CCTTGGTCGTTTTGATTTTTGT	5081–6304
	AAGATAGCACAAACATCCCAAAG	
E	GGCAGTCACATAATTAAAGACACAT	6061–7292
	TGGACCTCCTCATAAAACACAT	
F	ATTCCGGTGATGTGTTTTATGAGGAGGT	7262–10,030
	ACATCACCATCCTGGACYTGTTGAAG	
G	TTACCACACCCACTTCCACCAGCATTG	9604–12,066
	AGAGGCTTTGCATAGACCCCATTTCATTTCAATTCA	
H	TGAATTGAAATGAAATGGGGTCTATGCAAAGCCTCT	11,973–14,584
	CGGCATCTGGAGGTGATGAATTTCCAGGTTTCTAT	
I	CCTTCGGGTACATGACATTCGT	14,543–15,347
	TTTTTTTTTTTTTTTTTTTTTTTTTTTAATTWCGG	

### Genetic variation analysis of GSWW/2015 strain

The correct genome sequence of GSWW/2015 strain was obtained by incorporation of the consistent sequencing results of different colonies of different fragments. The whole genome of GSWW/2015 was aligned with 34 PRRSV reference strains (Table 2) and a phylogenetic tree was constructed using MEGA5.2 based on the neighbor-joining method. Bootstrap values were calculated on 1000 replicates. The variation of different coding and non-coding regions of GSWW/2015 was analyzed by comparison with corresponding parts of VR-2332, LV, CH-1a and HUN4 strains by ClustalW in Lasergene software (Version 7.1). The amino acid variation of part of Nsp2, GP3 and GP5 was shown in detail by aligned with 34 reference strains.

**Table 2:** Thirty-four PRRSV reference strains used for sequence alignment

No.	Strains	GenBank No.	No.	Isolates	GenBank No.
1	VR-2332	PRU87392	18	CG	EU864231
2	MLV	AF066183	19	GDQY2	GU454850
3	CH-1a	AY032626	20	SHH	EU106888
4	BJ-4	AF331831	21	08HuN	GU169411
5	S1	DQ459471	22	08SDWF	GU168569
6	HB-1sh	AY150312	23	GDBY1	GQ374442
7	HB-2sh	AY262352	24	KP	GU232735
8	CH2002	EU880438	25	WUH2	EU678352
9	CH2003	EU880440	26	WUH3	HM853673
10	CH2004	EU880439	27	YN2008	EU880435
11	HN1	AY457635	28	GS2008	EU880431
12	CC1	EF153486	29	XL2008	EU880436
13	Em2007	EU262603	30	09HUB5	GU168568
14	CH-1R	EU807840	31	09HUB7	GU168567
15	HUN4	EF635006	32	HLJHL	HM189676
16	JXA1	EF112445	33	SX-1	GQ857656
17	JXwn06	EF641008	34	SX2009	FJ895329

### The pathogenicity of PRRSV GSWW/2015 strain to pigs

To investigate the pathogenicity of PRRSV GSWW strain, 26 four-week-old piglets (Large White) approximately 20 kg were selected for this challenge experiment. We did not perform a sample size calculation. All pigs came from a pig farm in a mountain village in Zhuanglang county of Gansu province where PRRS has not been reported before. All pigs were twice detected to be negative for antibody against PRRSV. All pigs were transported to our Animal Biosafety Level 3 Laboratory in Lanzhou Veterinary Research Institute. The pigs were divided evenly into 2 groups and raised in 2 rooms approximately 20 square meters for one week before challenge. Nine and ten piglets in 2 rooms were intranasally challenged with 10^8^ copies of GSWW/2015 virus, and seven piglets were kept in the same room as contact-infection control. Whole blood samples were collected before challenge and at 4, 7, 14, 21, 28, 35, 42, 49, 56, 63 days post infection (DPI) for viremia detection. The serum samples were also collected before infection and at every week point until 125 DPI for antibody detection. At the end of experiment, the piglets were anaesthetized intramuscularly with 15 mg/kg ketamine (Ursotamin, Serumwerk-Bernburg AG, Bernburg, Germany) and 2 mg/kg azaperon (Stresnil, Janssen-Cilag GmbH, Baar, Switzerland) prior to euthanization by the i.v. administration of 200 mg/kg pentobarbital (Pentobarbital 10%, NAF Apotek, Oslo). Then the piglets were humanly bled to death.

### Quantitative real-time PCR of the viremia levels

The whole blood samples were used to extract the viral RNA by Trizol, and the virus RNA copies were detected by Real-time RT-PCR method as described by Liu et al. [12] (2010, in Chinese). The quantitative PCR primers and probe are as following: Forward: 5’-CGGCAAATGATAACCACGC-3′, Reverse: 5’-TTCTGCCACCCAACACGAG-3′; TaqMan probe: 5’-FAM-TGTGCCG TTRACCGTAGTGGAGCC-TAMRA-3′.

### PRRSV specific antibody detection by LSI ELISA

PRRSV antibody in serum samples were detected by a commercial ELISA kit (LSI Vet™ Porcine PRRS/US-Serum) from LSI company of France. This kit is based on the principle of an indirect ELISA. The assays were performed according to the manufacturers’ specifications. Antibody value (IRPC value) was expressed as the following formula: IRPC = (OD sample-OD NC)/ (OD PC-OD NC) × 100%. If the IRPC value is greater than 0.2, the sample is judged to be positive for PRRSV antibody; Otherwise, the sample is judged to be negative for PRRSV antibody.

## Results

### Virus isolation and identification

**Fig. 1:**
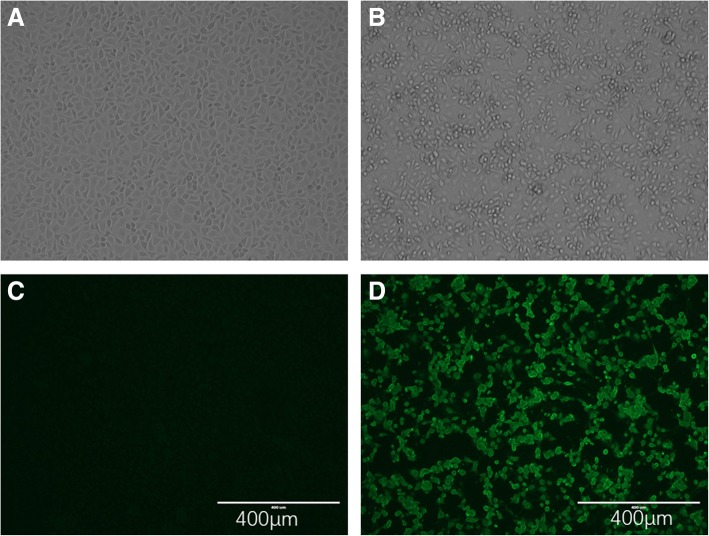
Isolation and Identification of PRRSV virus in Marc-145 cells. **a** showed normal cell; **b** showed the CPE after inoculation of viral material; **c** showed the normal cell after incubation with SR30 monoclonal antibody by IFA; **d** showed the positive results after reaction with SR30 antibody by IFA

The causative agent was confirmed to be PRRSV by RT-PCR and IFA. One specific DNA band of approximately 375 bp was amplified as expected. After inoculation of Marc145 cells with the viral material prepared with lung tissue, the typical CPE of PRRSV infection can be observed within 72 h after 2 passages (Fig. 1a, b). PRRSV antigen was also detected in Marc145 cells after incubation with SR30 Mab recognizing Nucleoprotein N (Fig. 1a-d). The results indicated the PRRSV strain was isolated by direct inoculation of Marc-145 cells with viral material. The virus strain was designated as PRRSV GSWW/2015.

### Full-length genomic sequence of PRRSV GSWW/2015

The full-length genome of GSWW/2015 was 15,319 nucleotides in length, excluding the poly (A) tail. The sequence identity between GSWW/2015 and other 4 representative PRRSV strains are listed in Table 3. The complete genome of GSWW/2015 strain shares 93.5, 96.9, 88.5 and 60.6% identity with CH-1a (the first PRRSV strain isolated in China in 1996), HUN4 [18] (a representative strain of HP-PRRSV), VR-2332 (NA-type) and LV (EU-type), respectively. The results indicate that GSWW strain belongs to the NA-type. The 5’-UTR, 3’-UTR, ORF1a, ORF 1b exhibited 62.0–99.5%, 74.6–98.4%, 57.3–96.3% and 63.3–96.9% sequence identity with 4 different reference strains, respectively (Table 3). Nsp2 was the most variable non-structural protein with 37.5–93.0% amino acid identity compared with 4 reference strains. GP3 and GP5 exhibited 55.7–94.5% and 58.2–95.5% amino acid identity respectively, indicating they were the most variable structural proteins. The N protein of GSWW strain was identical with HUN4 strain. The M protein was the most conserved structural protein sharing 80.3–99.4% amino acid identity with other 4 reference strains (Table 3).

**Table 3:** Homology of the different parts of genome and proteins of GSWW/2015 compared with representative strains of PRRSV

	HUN4	CH-1a	VR-2332	LV
Complete	96.9	93.5	88.5	60.6
5’ UTR	99.5	97.3	92.0	62.0
ORF1a	96.3	92.2	86.3	57.3
ORF1b	96.9	94.4	90.2	63.3
ORF2	98.1	95.3	93.3	66.0
ORF3	97.0	94.1	87.8	64.8
ORF4	97.4	95.0	89.2	67.4
ORF5	97.0	92.9	87.7	64.1
ORF6	99.6	97.3	95.2	69.5
ORF7	99.5	96.2	93.5	68.7
3’UTR	98.4	95.3	92.0	74.6
NSP2	93.0	84.3	73.0	37.5
GP2	96.1	93.8	92.6	61.8
GP3	94.5	90.6	85.0	55.7
GP4	97.8	97.2	89.9	70.8
GP5	95.5	90.5	87.5	58.2
M	99.4	97.7	97.7	80.3
N	100.0	95.1	95.1	65.0

Notes:Different ORF and UTR regions are analyzed based on the nucleotide sequence. NSP2, GP2–5, M and N protein are analyzed by deduced protein sequences

### Phylogenetic analysis

**Fig. 2:**
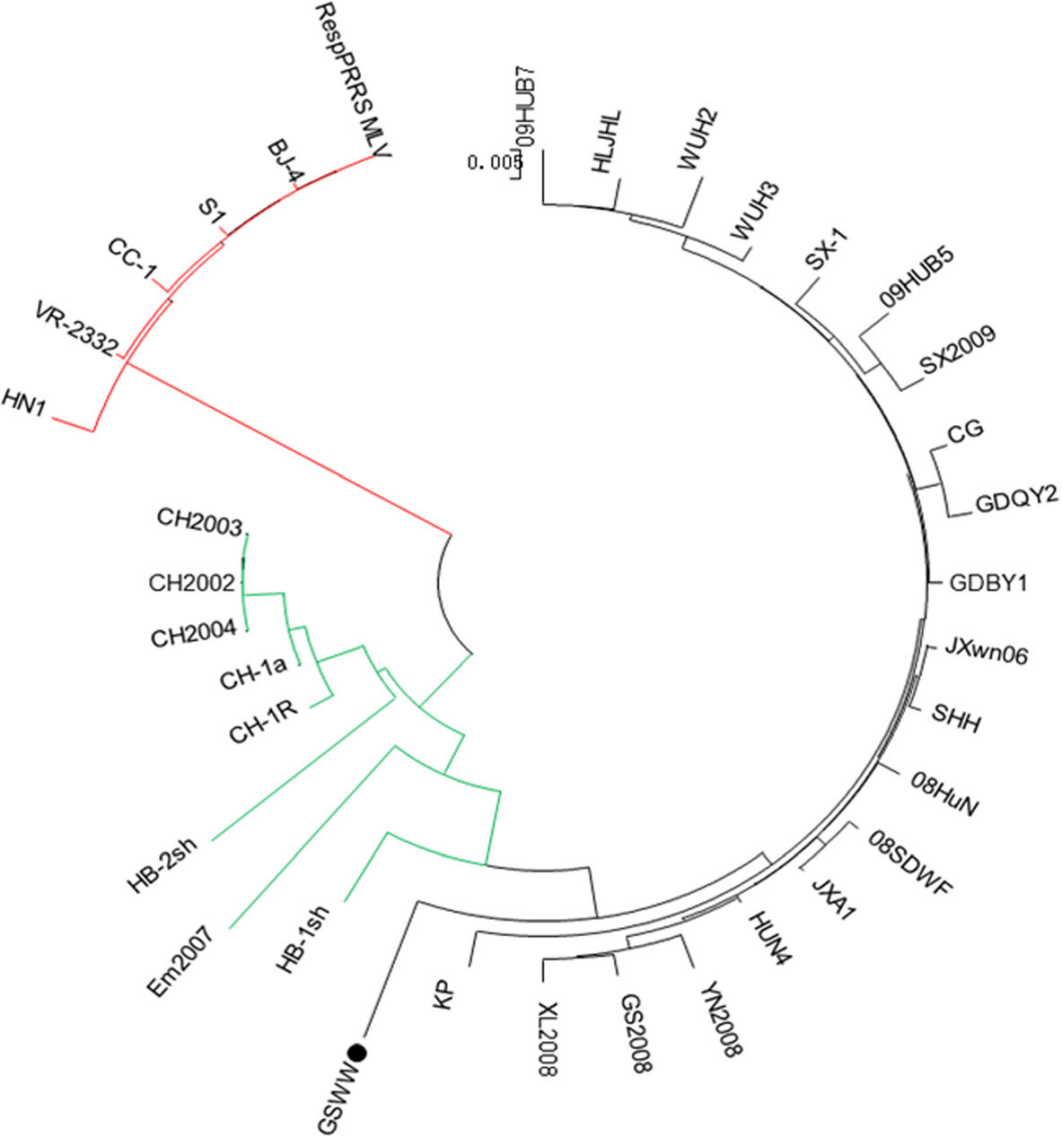
Phylogenetic tree drew by comparing the whole genome of GSWW/2015 with 34 reference PRRSV strains by MEGA5.2. Three subgroups are indicated by different colors: subgroup 1 (red) includes 6 strains, subgroup 2 (green) includes 8 strains, subgroup 3 (black) includes 21 strains. The newly isolated strain GSWW belongs to subgroup 3 as indicated by a black dot

A phylogenetic tree was constructed based on the complete genome sequences of GSWW and 34 PRRSV reference strains (Fig. 2). Phylogenetic analysis revealed that the NA-type PRRSV was divided into three subgroups. Subgroup 1 comprised 6 strains: VR-2332, RespPRRS MLV, BJ-4, S1, HN1 and CC1. Subgroup 2 included CH-1a, Em2007, HB-1sh, HB-2sh, CH-1R, CH2002, CH2003 and CH2004. Subgroup 3 consisted of 21 HP-PRRSV strains including GSWW/2015 strain, and the representative strains were HUN4, JXA1 and JXwn06.

### Amino acid variation of NSP2, GP3 and GP5 proteins

**Fig. 3:**
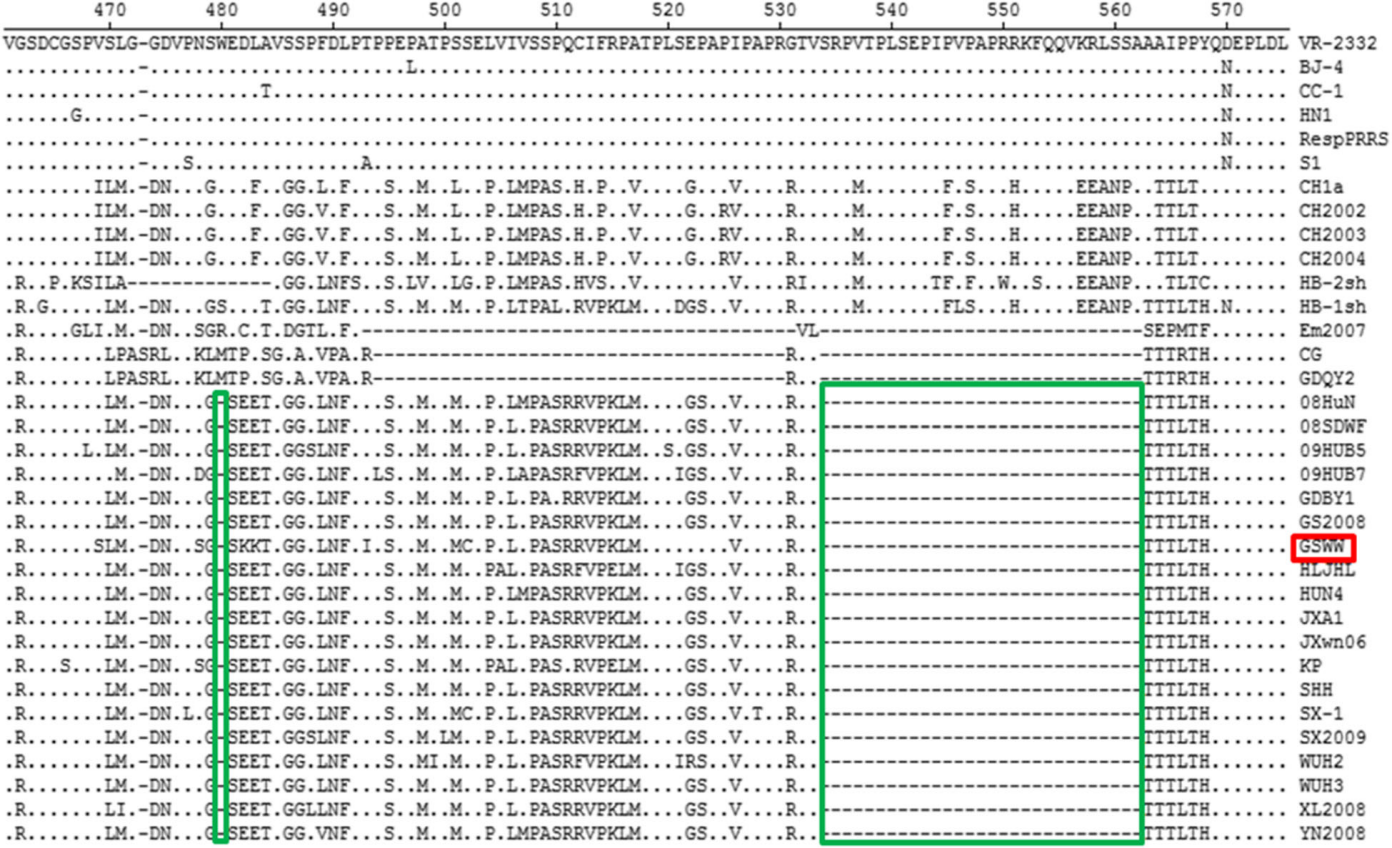
High variable regions in NSP2 of GSWW/2015 compared with 34 reference PRRSV strains. A discontinuous deletion of 30-aa (positions 480 and 534–562) were indicated by two green boxes in HP-PRRSV emerged in China in 2006

To explore the genetic variation in specific proteins, amino acid sequences of Nsp2, GP3 and GP5 were aligned with 34 PRRSV reference strains (Fig. 3). The NSP2 region of GSWW/2015 strain shows a discontinuous deletion of 30-aa at positions 480 and 534–562, which is a typical feature of HP-PRRSV strains emerged in China since 2006 [17]. The amino acid sequence of NSP2 shows 91.6–93.2% similarity with other HP-PRRSV strains indicating a remarkable genetic variation in this protein.

**Fig. 4:**
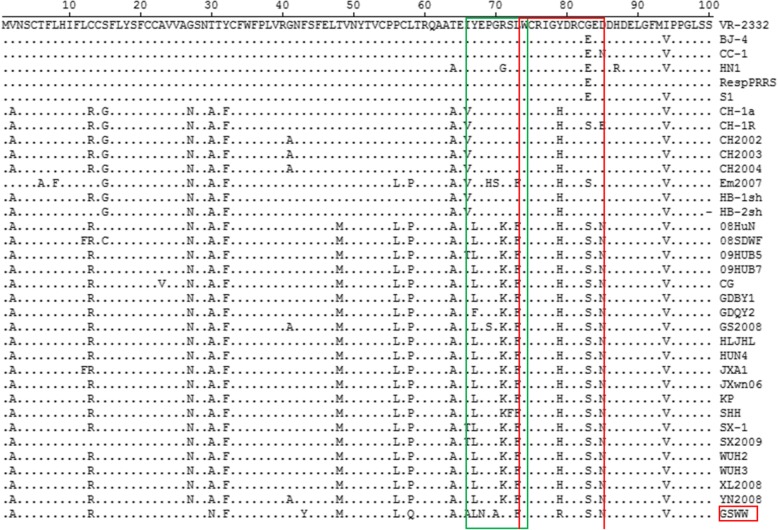
GP3 protein of GSWW/2015 compared with 34 reference PRRSV strains. Two antigenic epitopes (^67^YEPGRSLW^74^ and ^74^WCRIGHDRCGED^85^) in GP3 were indicated in green and red boxes

Two antigenic epitopes (^67^YEPGRSLW^74^ and ^74^WCRIGHDRCGED^85^) in GP3 had been described previously [20]. GP3 of GSWW/2015 strain shows some accumulated mutations in these two epitopes. Two unique amino acid mutations (E^68^N and G^70^A) in the epitope (^67^YEPGRSLW^74^) were observed compared with other HP-PRRSV strains, while another two amino acid mutations (Y^67^L and L^73^F) were the same with other HP-PRRSV strains. In the epitope (^74^WCRIGHDRCGED^85^), GSWW/2015 strain shows one specific amino acid mutations (Y^79^H). Some other specific mutations can also be observed at positions A/T^30^N, F^43^Y and L/P^58^Q (Fig. 4). The influence of these changes on the antigenicity of GP3 need to be further investigated.

**Fig. 5:**
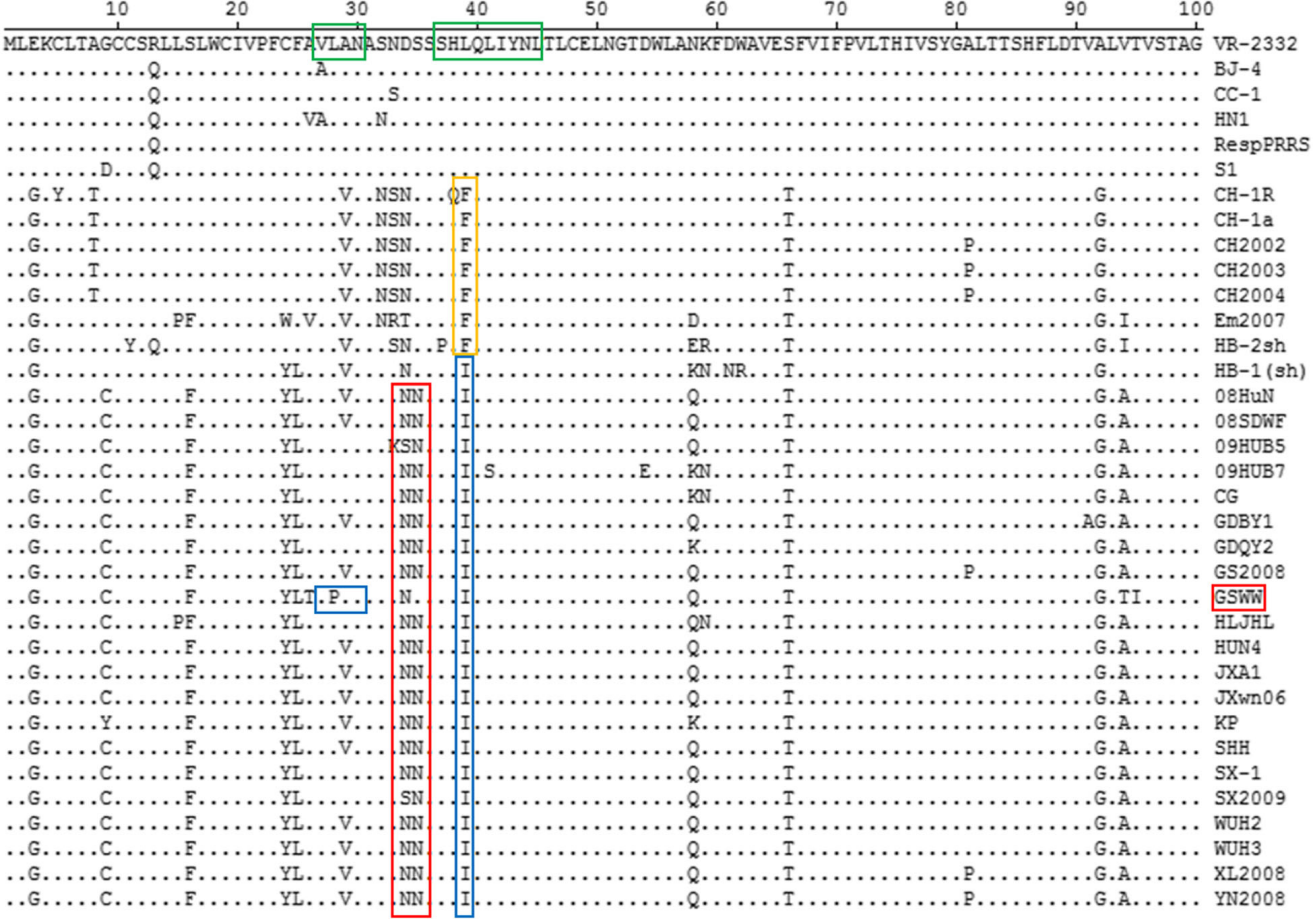
Part of GP5 protein of GSWW/2015 compared with 34 reference PRRSV strains. The primary neutralizing epitope (PNE) (^37^SHF/LQLIYNL^45^) and the decoy epitope (^27^V/ALVN^30^) are indicated by two green boxes. The putative N-linked glycosylation sites are indicated by a red box. The decoy epitope of GSWW/2015 strain has one unique amino acid mutation (L^28^P) and one N-linked glycosylation site is absent at position 35 compared with other HP-PRRSV strains

The amino acid mutations were described here for GSWW/2015 in the primary neutralizing epitope (PNE) (^37^SHF/LQLIYNL^45^) and the decoy epitope (^27^V/ALVN^30^) of GP5 protein [16]. Two amino acid mutations (A/V^26^T, L^28^P) specific for GSWW/2015 were observed in the decoy epitope region compared with other HP-PRRSV strains and subgroup 1 and 2 isolates. One N-linked glycosylation site (N-X-S/T) is absent at position 35 (N^34^S) of GSWW/2015 strain compared with other HP-PRRSV (Fig. 5).

### The pathogenicity of PRRSV GSWW/2015 strain to pigs

**Fig. 6:**
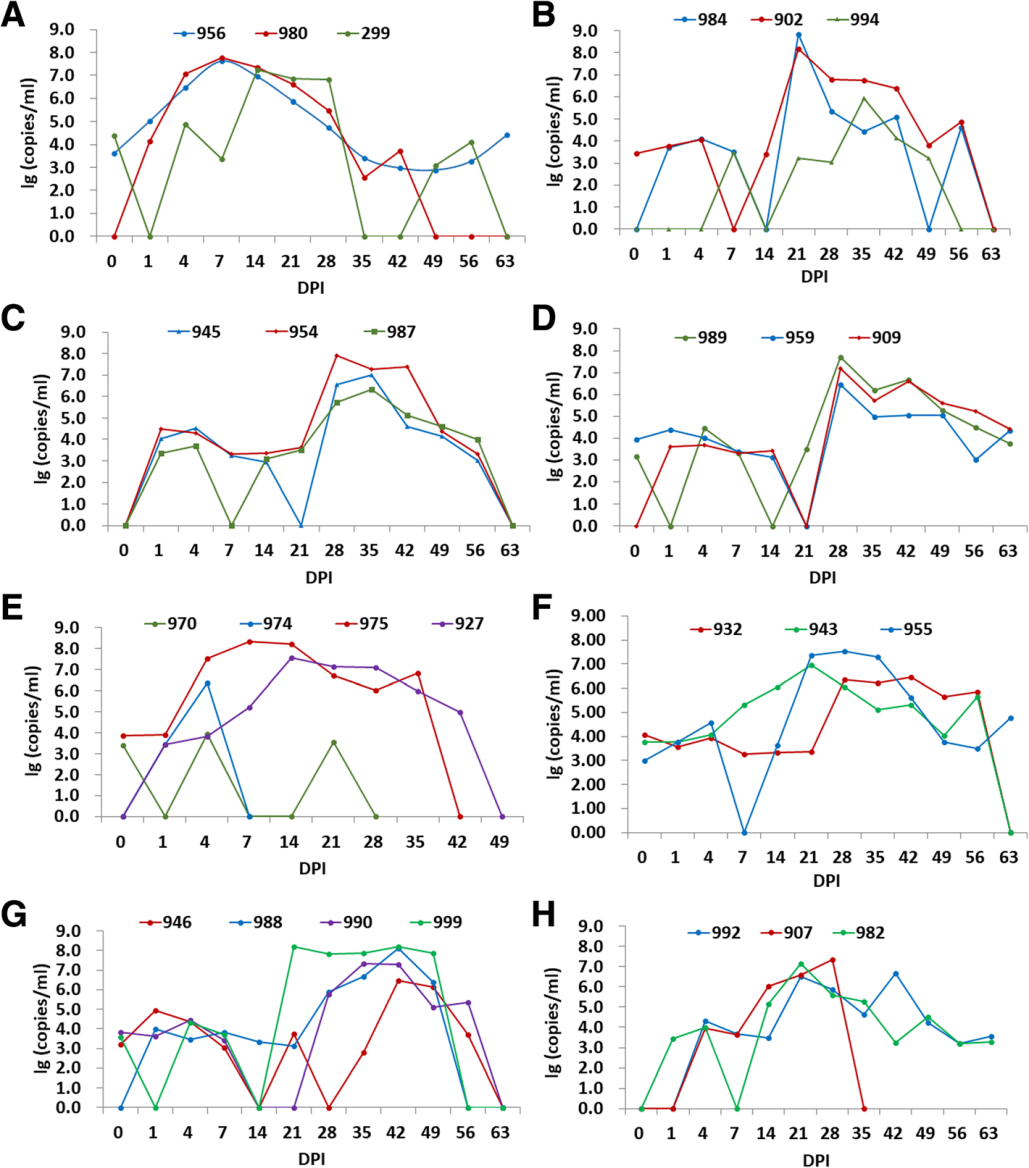
Viremia levels in blood detected by qRT-PCR. **a**-**f** showed the results of pigs infected by airway; **g**, **h** showed the results of 7 contact-infected pigs

**Fig. 7:**
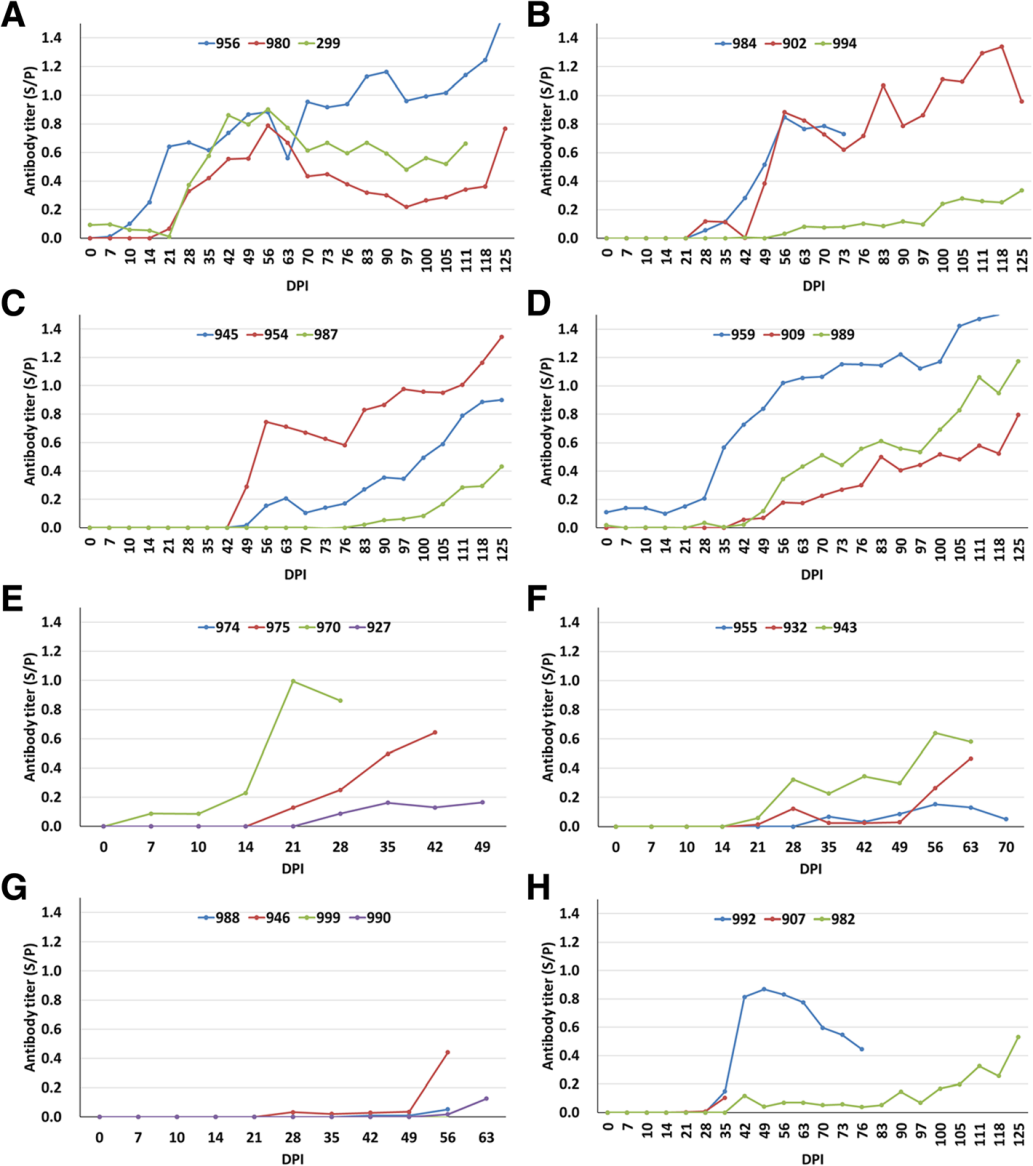
Antibody responses to PRRSV detected by LSI ELISA kit. **a**-**f** showed the results of pigs inoculated by airway; **g**, **h** showed the results of 7 contact-infected pigs. Antibody titer greater than 0.2 were judged to be positive for PRRSV antibody

Nineteen pigs were challenged by airway inoculation of PRRSV GSWW/2015 strain. Two infected piglets died early without obvious clinical signs. One pig (No.974) showed viremia on day 4 and died on day 7 (Fig. 6e). One pig (No.970) died on day 28 without obvious viremia (Fig. 6e) but seroconversion was observed after 14 DPI (Fig. 7e). The other 24 piglets gradually appeared clinical signs of typical PRRS, such as fever, lethargy and emaciation, some showed blue ears, anhelation. Five pigs (No. 956, 980, 975, 927 and 299) showed peaked viremia at 7 and 14 DPI (Fig. 6a-h), and the other 19 pigs showed peaked viremia at 21 DPI (7 pigs, No. 984, 902, 955, 943, 999, 982 and 992), 28 DPI (6 pigs, No. 907, 932, 989, 959, 909 and 954), 35 DPI (4 pigs, No. 945, 987, 994, 990) and 42 DPI (2 pigs, No. 946, 988), respectively. Seven contact-infected pigs showed peaked viremia after 14 DPI. The viremia lasted for one month in most pigs, however virus activity could be detected in herd level during the whole period of experiment. Fourteen piglets (No. 907, 999, 975, 927, 946, 988, 932, 943, 990, 955, 984, 992, 299 and 959) showed high level of viremia and died gradually at 35 to 118 DPI (Figs. 6 and 7). The rest 10 pigs (No. 956, 980, 902, 994, 945, 954, 987, 989, 909 and 982) were anaesthetized and humanely bled to death at the end of the experiment. Dead pigs were proceeded pathologic anatomy and observed the typical pathological changes of PRRS, such as consolidation of lungs, swelling and diffusely hemorrhage in lungs and lymph nodes.

### The antibody response to PRRSV detected by a commercial ELISA kit (LSI ELISA)

The antibody responses to PRRSV were shown in Fig. 7 for all pigs at different time point. The earliest seroconversion was observed in two pigs No. 956 and 970 at 14 DPI (Fig. 7a, e), and then 5 pigs (No. 299, 980, 959, 975 and 943) (Fig. 7a, d, e, f) at 28 DPI. There were another 12 pigs (No. 984, 992, 902, 954, 932, 946, 989, 945, 909, 982, 987 and 994) gradually became serological positive for PRRSV antibody during 42~ 125 DPI (Fig. 7a-h). Seven pigs (No. 974, 927, 955, 988, 999, 990 and 907) with high level viremia after virus infection did not show seroconversion for PRRSV antibody because of death before 70 DPI including 4 contact-infected pigs (Fig. 7g, h).

## Discussion

PRRS is considered one of the most important diseases affecting Chinese pig industry. In 1995, PRRSV was first isolated in China. Several PRRSV strains were subsequently isolated and sequenced in the following years, including BJ-4, HB-1(sh)/2002 and HB-2(sh)/2002. In 2006, a large-scale HP-PRRS outbreak was reported in China with characteristics of high morbidity and mortality. Studies indicated that HP-PRRSV originated from CH-1a-like virus and experienced gradually genetic variation and accumulation in genomic sequences [2]. HP-PRRSV rapidly became the dominant strains prevailing in the field.

In this study, we isolated a new highly pathogenic PRRSV strain (designated as GSWW/2015) circulated in north-western region of China as confirmed by RT-PCR, IFA and animal challenge experiments. The complete genome was sequenced and compared with 34 reference strains. Phylogenic analysis showed that the GSWW/2015 belongs to subgroup 3 of NA-type PRRSV as HP-PRRSV strain. Subgroup 1 and 2 isolates were classical PRRSV. VR-2332 and CH-1a were the representative strains of subgroup 1 and 2, respectively. In addition, we analyzed the genetic variation in different regions of genome by comparison of GSWW/2015 with four representative strains. NSP2 is the highest variable region, and then GP3 and GP5 proteins (Table 3). It is still not clear of the mechanism for PRRSV to maintain this highly variable feature in NSP2 region.

NSP2 experiences remarkable genetic variation and contains natural insertions and deletions in the hypervariable region [8, 14]. Given the tolerance of insertions and deletions in the nonessential region, NSP2 is considered a potential candidate gene for genetic modifications [9]. The natural deletion of 12 amino acids in NSP2 was first found in HB-2(sh)/2002 strain [11]. The 30-aa deleted region of HP-PRRSV contained B-cell epitopes [7] and T-cell epitopes [6]. The deletion of 30-aa in NSP2 was considered the genetic marker of HP-PRRSV, but it was not related to the increased virulence [21]. It is similar with other HP-PRRSV strains for GSWW/2015 strain to contain a discontinuous deletion of 30 amino acids in NSP2.

GP5 is a major inducer of neutralizing antibodies in vivo. The decoy epitope in GP5 was hypervariable and highly immunodominant, while the PNE was conserved among PRRSV isolates and was not immunodominant. Thus, antibody to the decoy epitope is dominant and responses are early after PRRSV infection, while antibody to PNE is delayed or at a very low level [16]. Sequence alignments showed that the GP5 decoy epitope of GSWW/2015 strain had one unique amino acid mutation (L^28^P), which is different from other HP-PRRSV strains listed in this paper. In the PNE, residues ^38^HLQLIYN^44^ were considered as the main recognition site for neutralizing antibodies. The residue of L39 in subgroup 1 changed to I/F39 in subgroup 2 and HP-PRRSV including GSWW/2015 strain, which may influence the binding of neutralizing antibodies. In addition, several putative N-linked glycosylation sites were in the ectodomain of GP5. N44 glycosylation site located in the PNE is critical for the recovery of infectious PRRSV. N33 and N51 mutations enhanced viral sensitivity to neutralization antibody. GSWW strain had one amino acid mutation at position 35 (N^35^D) which is different from other HP-PRRSV strains and similar with earlier PRRSV stains in subgroup 2. It had been reported that the glycosylation was related with viral immune evasion and minimized neutralizing antibody response against the PNE [1].

GP3 is the most variable structural protein as showed by comparing GP3 protein of GSWW/2015 with other representative PRRSV strains. Although antibody to GP3 is produced at a very low level, it may play an important role in neutralization and clearance of virus [4]. There are more amino acids mutations observed in GP3 of GSWW/2015 strain compared with other HP-PRRSV strains, including E^68^N, G^70^A, K^71^R in epitope ^67^YEPGR/KSLW^74^ and H^79^R in epitope ^74^WCRIGY/HDRCGED/N^85^ [20], as well as changes at A/T^30^N, F^43^Y, L/P^58^Q. The biological meaning of these mutations need to be further illustrated.

Considering the fundings, size of the pig farm and capacity of our P3 lab, we thought 26 piglets were enough to investigate the characteristics of the virus. Pig challenge experiment showed that GSWW/2015 is a highly pathogenic PRRSV strain. After challenge, most piglets showed fever and obvious clinical signs, such as listlessness and anorexia. The clinical signs became more severe over the next few days with lethargy and emaciation. Fourteen piglets died gradually at 35 to 118 DPI, which showed high level of viremia and developed enlarged tonsil, swelling and diffuse consolidation and hemorrhage in lung and lymph nodes. Some piglets showed viremia early on day 1 (No.946 and 956), day 4 (No.299, 974, 975 and 980) and day 7 (No.943), while some showed viremia late on day 14 (No.907 and 982), day 21 (No.902 and 984 etc) and day 28 (No.945 and 954 etc). Contact-infected pigs (Fig. 7g, h) showed viremia later than airway inoculated pigs except for one pig No. 946. Most pigs inoculated by airway or contact-infected showed peaked viremia after 2–3 weeks of exposure. This may reflect a complex situation for PRRSV infection in pig herd. The viral titer in blood could reach up to 10^8^ copies/ml. The viremia lasted over a month individually, but overall the viremia lasted over two months because the piglets developed viremia at different time.

The antibody responses to PRRSV were overall delayed and not sufficient. Seven pigs that showed high level of viremia after infection did not show seroconversion for PRRSV antibody until death (Fig. 7g, h). Two pigs (No. 987 and 994) that showed peaked viremia at 28 and 42 DPI developed detectable antibody to PRRSV at 111 and 100 DPI (Fig. 7c, b). These results indicate that serological method seem not to be so sensitive for detection of PRRSV infection.

## Conclusions

GSWW/2015 strain is a highly pathogenic PRRSV strain circulated in north-western of China. This virus strain displays a unique discontinuous deletion of 30-aa in NSP2 and accumulated amino acids changes in GP3 and GP5, which might alter the antigenicity of the virus. Furthermore, GSWW/2015 strain can induce high level of viremia and persistent infection in individual and herd level. Antibody response to PRRSV is delayed compared with detection of viremia for diagnosis of virus infection. The correlation between long-lasting viremia and low level of antibody responses needs to be further investigated.

## Abbreviations

CPE: Cytopathic effect
EAV: Equine arteritis virus
HP-PRRSV: Highly pathogenic porcine reproductive and respiratory syndrome virus
IFA: Indirect immunofluorescent assay
LDV: Lactate dehydrogenase-elevating virus
NSP: Non-structural protein
PRRS: Porcine reproductive and respiratory syndrome
PRRSV: Porcine reproductive and respiratory syndrome virus
qRT-PCR: Quantitative real-time PCR
SHFV: Simian hemorrhagic fever virus
